# Supplementary figures and images for: Transcriptomic Analysis Reveals the Flavonoid Biosynthesis Pathway Involved in Rhizome Development in Polygonatum cyrtonema Hua
Source: Plants (Basel). 2024 May 31;13(11):1524. doi: 10.3390/plants13111524 (PMC11174788; doi:10.3390/plants13111524)

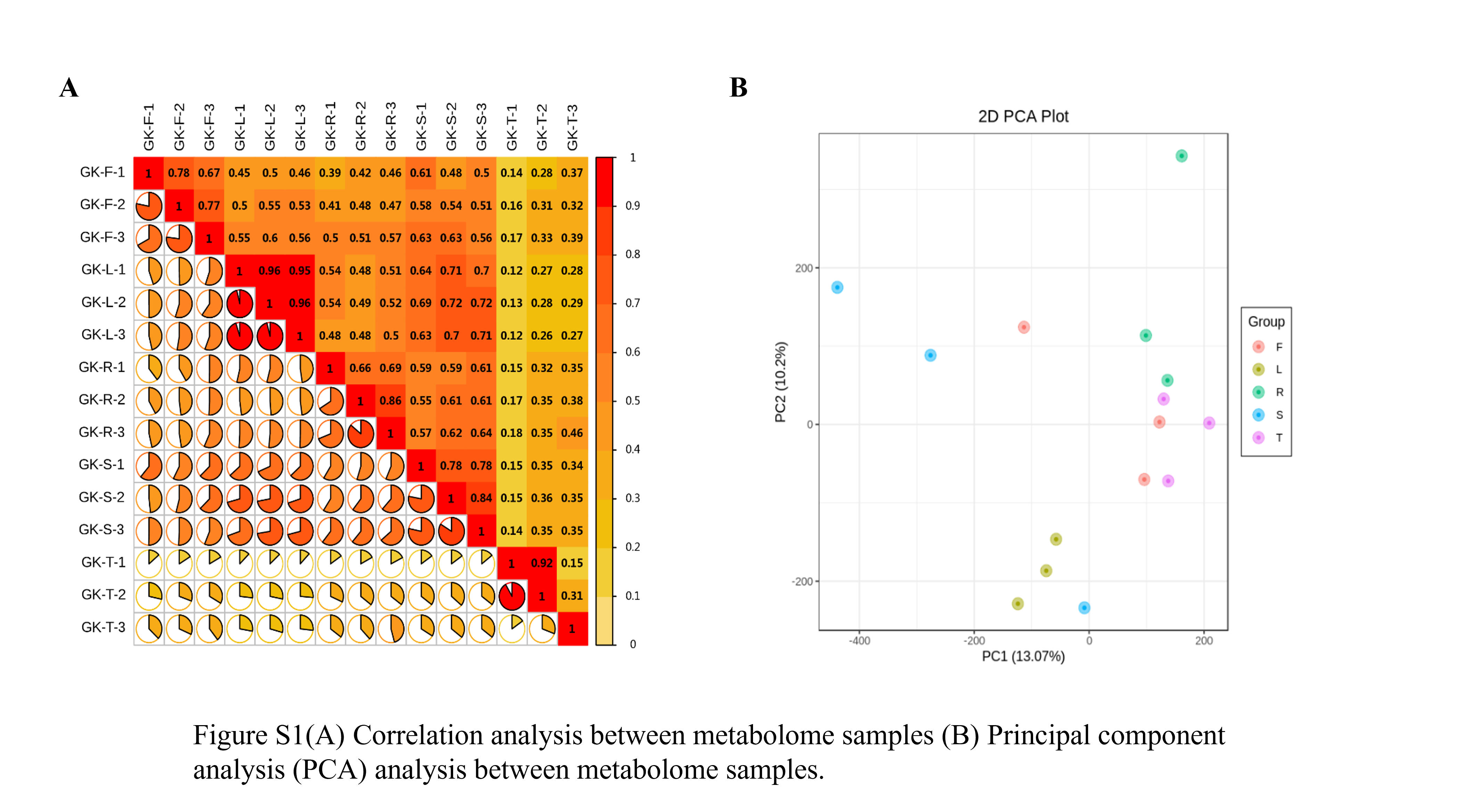

Supplement: Supplementary file 1 [file plants-13-01524-s001.zip › Figure S1.png]

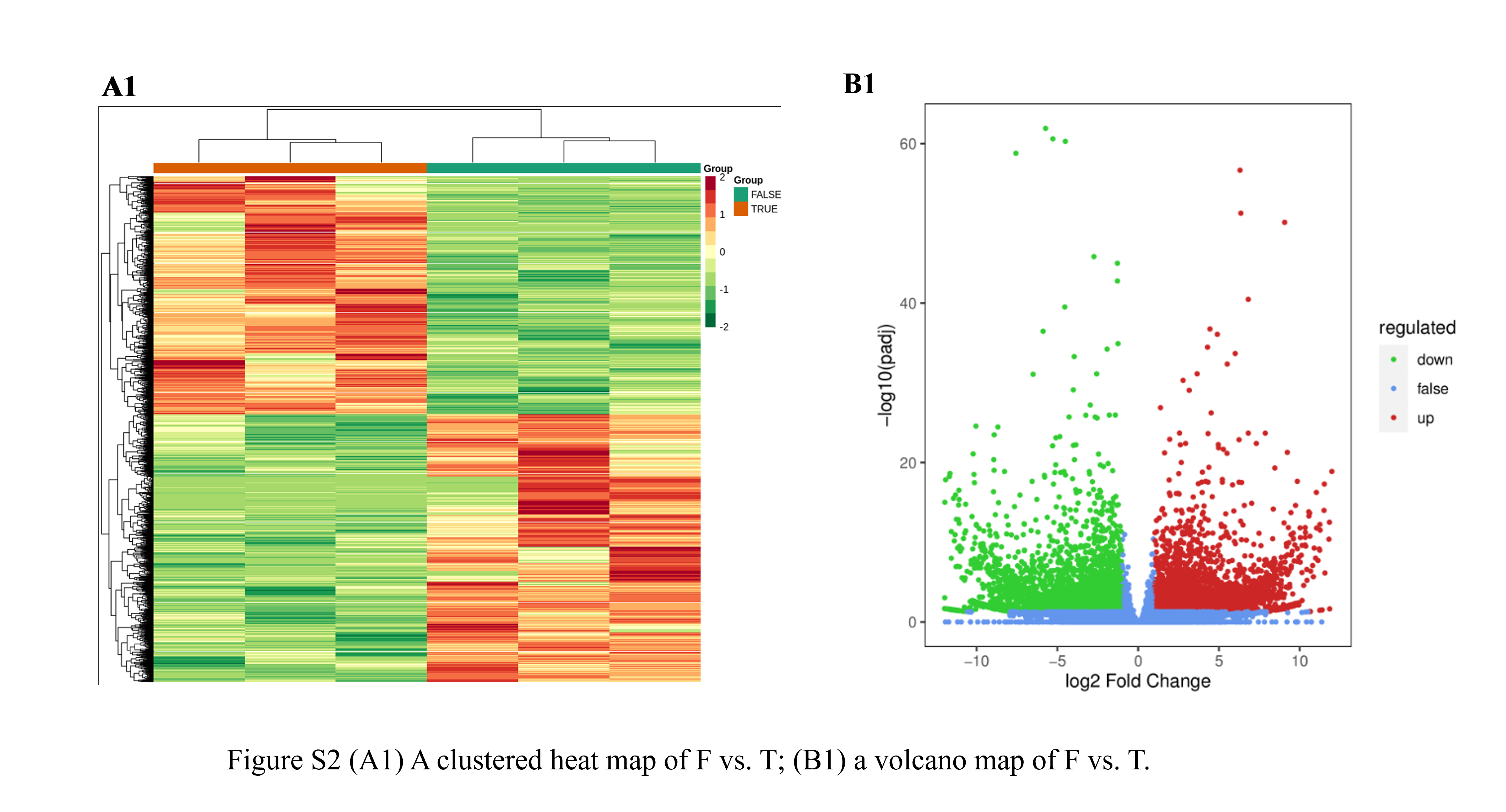

Supplement: Supplementary file 1 [file plants-13-01524-s001.zip › Figure S2(A1 and B1).png.png]

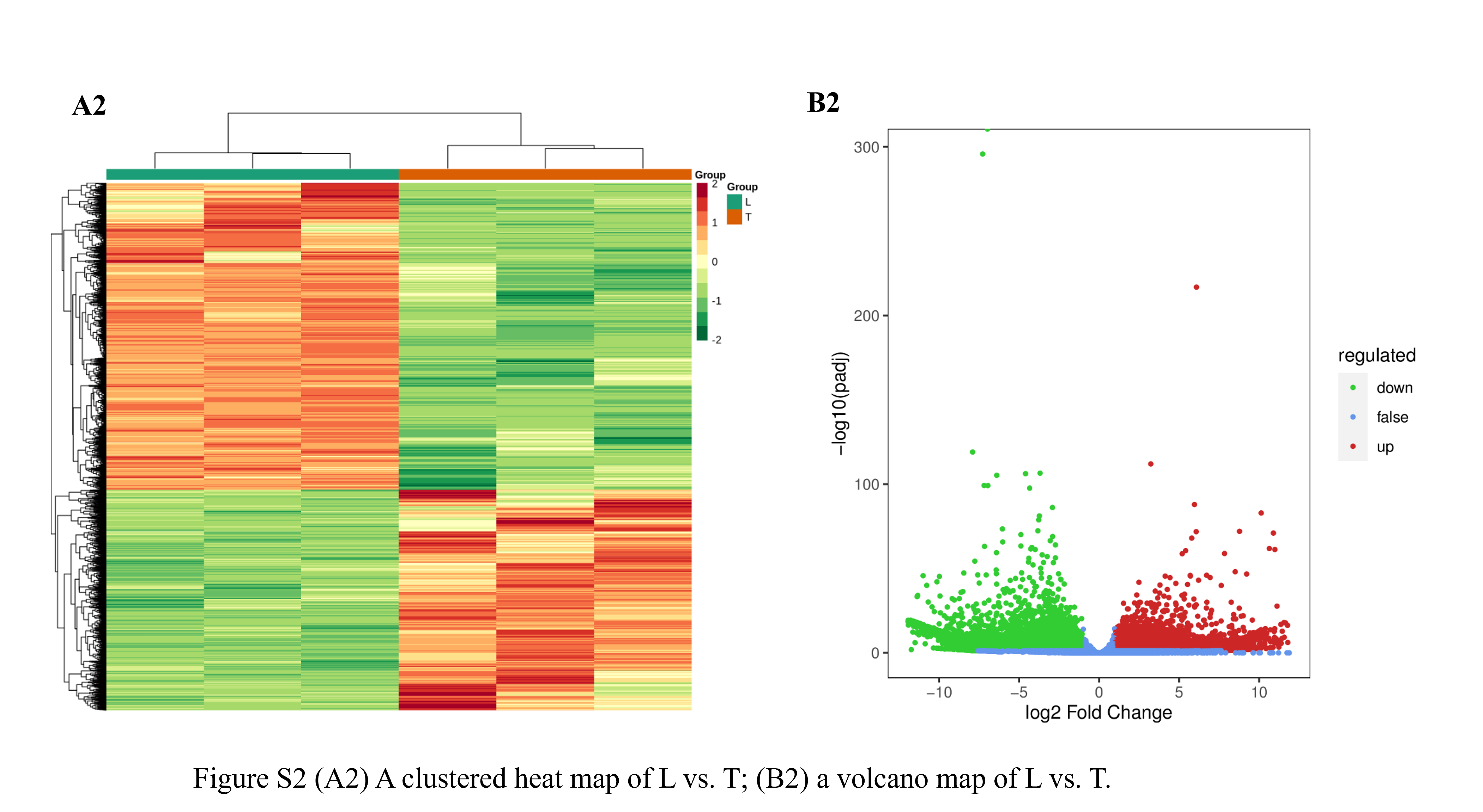

Supplement: Supplementary file 1 [file plants-13-01524-s001.zip › Figure S2(A2 and B2).png.png]

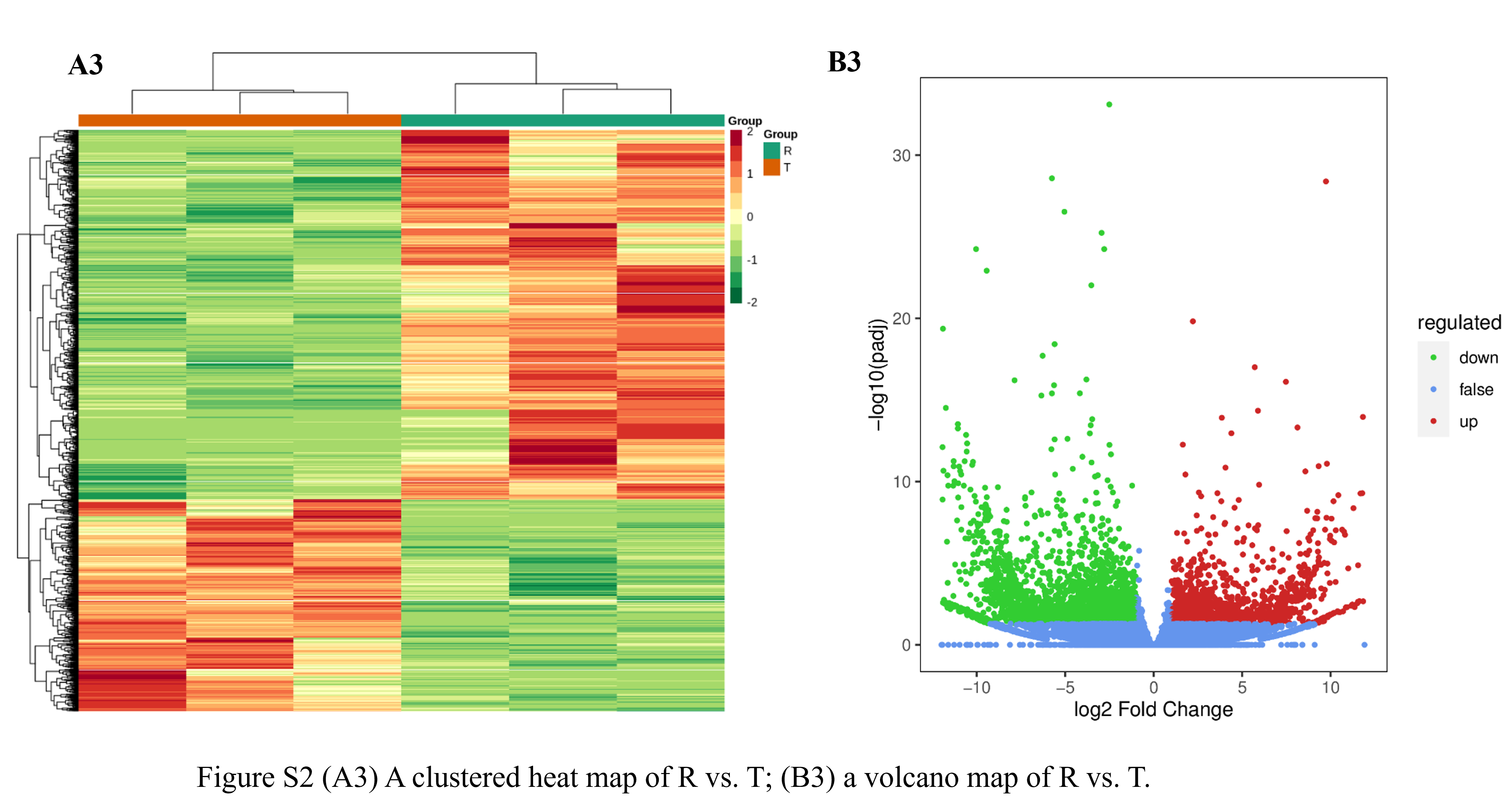

Supplement: Supplementary file 1 [file plants-13-01524-s001.zip › Figure S2(A3 and B3).png.png]

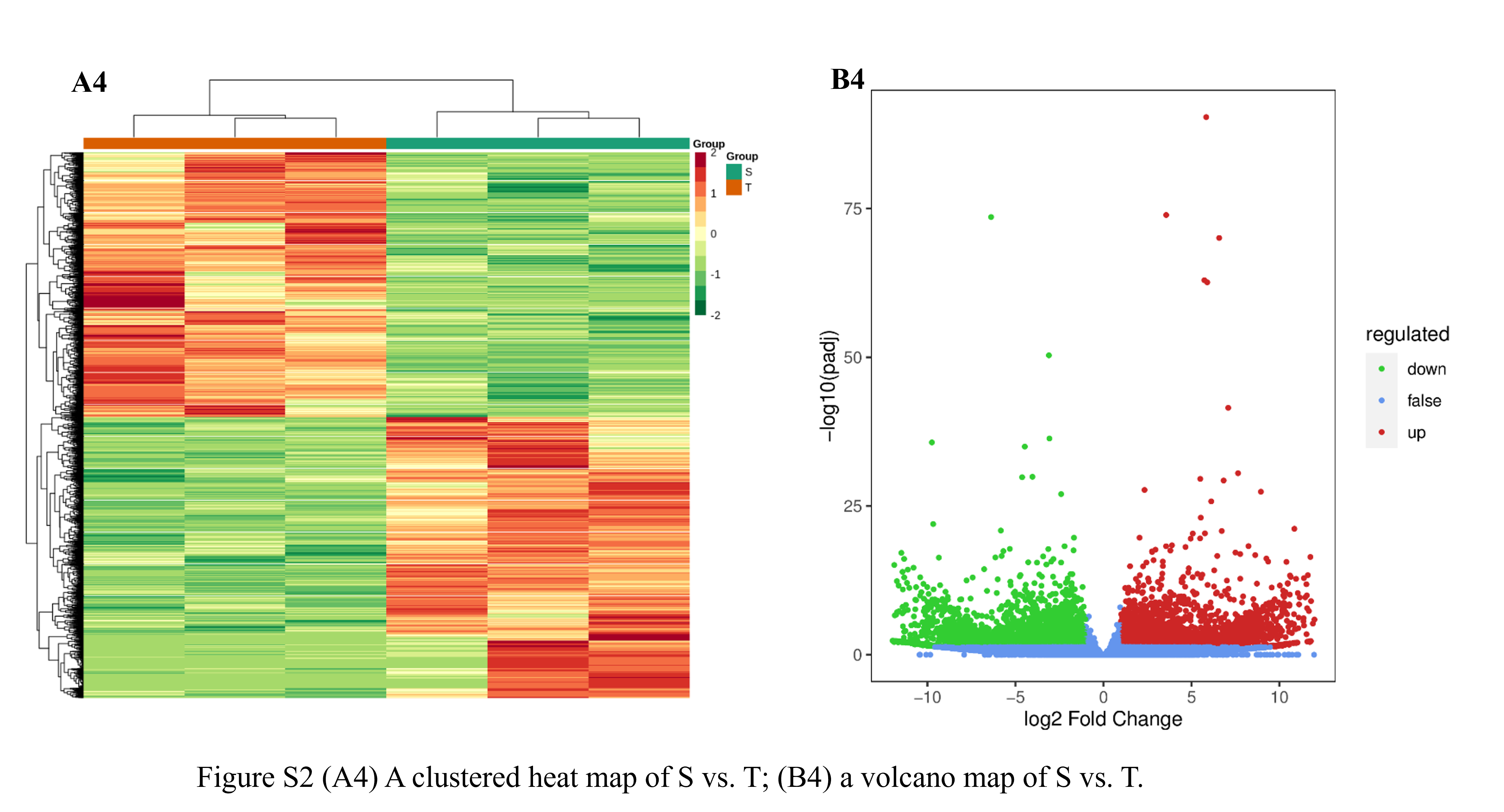

Supplement: Supplementary file 1 [file plants-13-01524-s001.zip › Figure S2(A4 and B4).png.png]

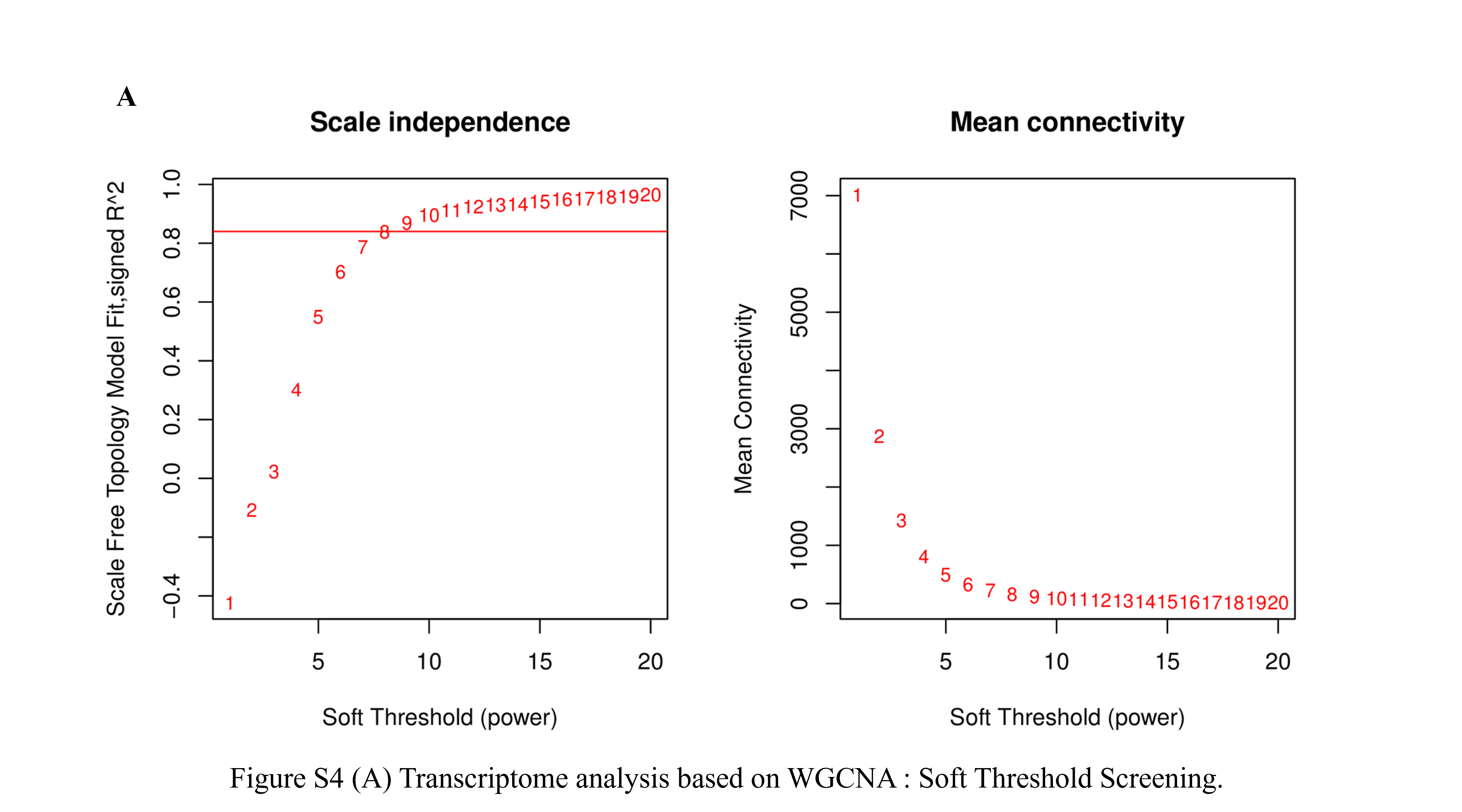

Supplement: Supplementary file 1 [file plants-13-01524-s001.zip › Figure S4(A).png]

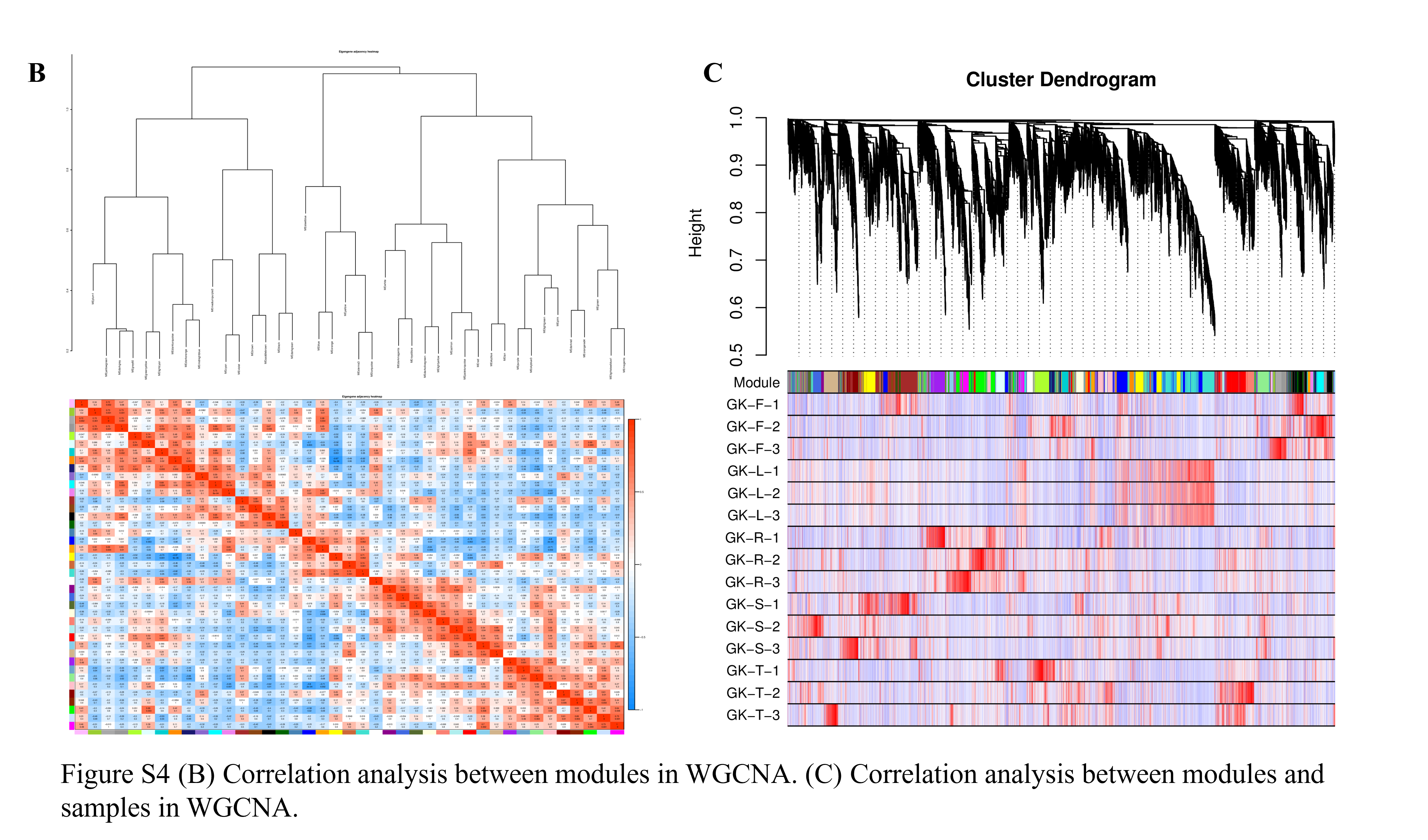

Supplement: Supplementary file 1 [file plants-13-01524-s001.zip › Figure S4ú¿B-Cú⌐.png]

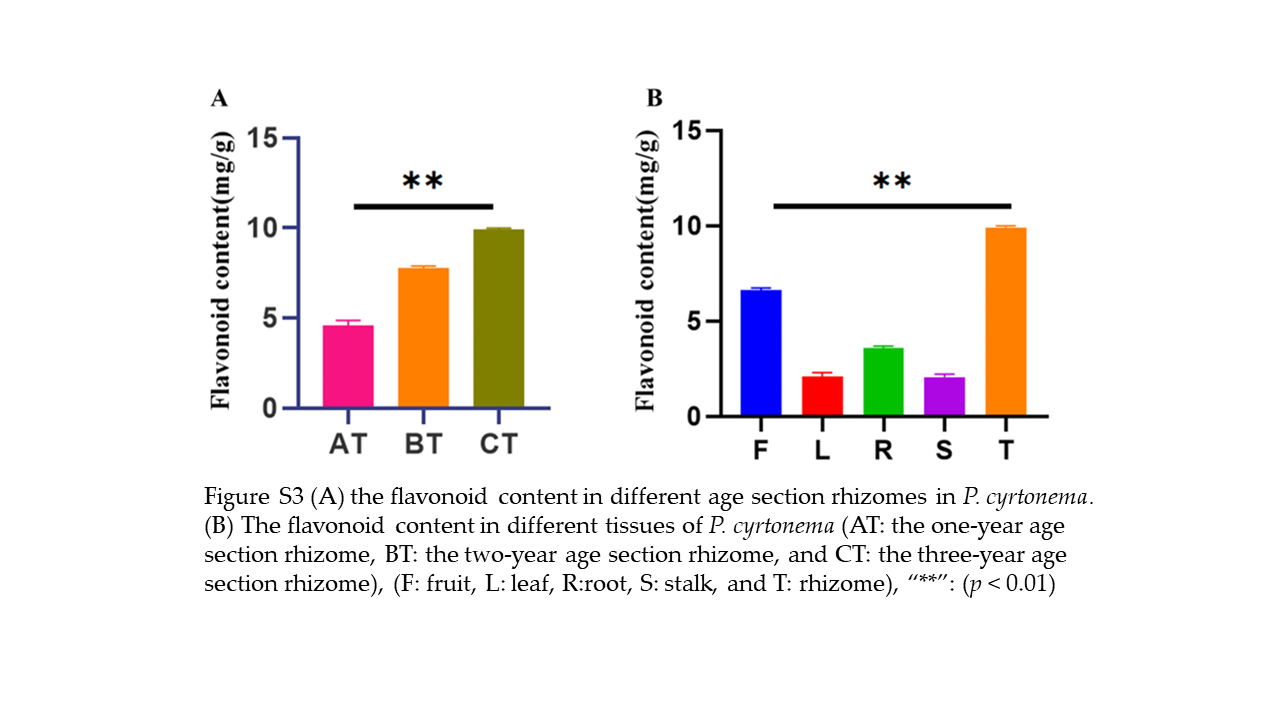

Supplement: Supplementary file 1 [file plants-13-01524-s001.zip › FigureS3(A-B).tif]
